# Objective and Subjective Voice Outcomes in Post-COVID-19 Dysphonia: A High-Speed Videoendoscopy Pre–Post Study

**DOI:** 10.3390/jcm14196861

**Published:** 2025-09-28

**Authors:** Joanna Jeleniewska, Jakub Malinowski, Ewa Niebudek-Bogusz, Wioletta Pietruszewska

**Affiliations:** Department of Otolaryngology, Head and Neck Oncology, Medical University of Lodz, 90-419 Lodz, Poland; joanna.jeleniewska@umed.lodz.pl (J.J.); ewa.niebudek-bogusz@umed.lodz.pl (E.N.-B.); wioletta.pietruszewska@umed.lodz.pl (W.P.)

**Keywords:** dysphonia, post-COVID-19 condition, high-speed videoendoscopy (HSV), videolaryngostroboscopy (VLS), larynx

## Abstract

**Background/Objectives**: The post-COVID-19 condition frequently includes dysphonia. We aimed to assess objective and subjective voice disorders and short-term responses to multimodal therapy in patients with isolated post-COVID-19 dysphonia. **Methods**: This retrospective, single-center pre–post study screened 244 post-COVID-19 patients; a subset of 14 with isolated dysphonia underwent standardized assessment at baseline and at 1-month follow-up. Patient-reported outcomes (Voice Handicap Index, VHI; Voice-Related Quality of Life, V-RQOL) and endoscopic evaluation were performed using videolaryngostroboscopy (LVS) and high-speed videoendoscopy (HSV) with kymographic analysis to quantify parameters describing vocal fold oscillations. The treatment included short-term systemic corticosteroids, inhaled corticosteroids, hyaluronic-acid inhalations, and structured voice therapy. **Results**: At baseline, HSV revealed signs of glottal insufficiency—irregular and asymmetric vocal fold motion, reduced amplitude and pliability, a disrupted mucosal wave, and an increased open quotient. At follow-up, HSV showed increased oscillation, amplitude, and cycle regularity with reduced left–right asymmetry and phase differences; phonovibrograms displayed clearer and more structured patterns. Perturbation indices decreased across jitter and shimmer measures, and the mean fundamental frequency was lower. Improvements in instrumental measures aligned with better VHI and V-RQOL scores. **Conclusions**: In patients with persistent dysphonia after acute SARS-CoV-2 infection, comprehensive ENT evaluation with instrumental laryngeal assessment is warranted. Short-term multimodal management was associated with improvements in both HSV-derived measures and patient-reported outcomes; confirmation in controlled studies is needed.

## 1. Introduction

Since late 2019, COVID-19 caused by the novel coronavirus SARS-CoV-2 has been associated with a wide spectrum of long-term health consequences. According to the WHO, approximately 6 in every 100 people who have COVID-19 develop the post-COVID-19 condition. Many different terms have been used in the literature to characterize this syndrome, such as long COVID, long-haul COVID, or post-COVID. The WHO defines the post-COVID-19 condition as symptoms lasting at least 2 months, starting usually 3 months after probable or confirmed SARS-CoV-2 infection, unexplained by an alternative diagnosis [1]. The National Institute for Health and Care Excellence (NICE) distinguishes acute COVID-19 (up to 4 weeks), ongoing symptomatic COVID-19 (4–12 weeks), and post-COVID-19 syndrome (>12 weeks). Current guidance emphasizes symptom-oriented, multidisciplinary management, with no proven pharmacological interventions for the condition itself to date [2]. In most published articles, these names are used interchangeably. In line with the recent WHO usage and ICD-10 coding, where the syndrome appears under U09.9 “Post-COVID-19 condition, unspecified,” we consistently use the term “post-COVID-19 condition” throughout this article [3]. Although initially associated with respiratory symptoms, the disease is now recognized as multisystemic in its impact. Over 200 different symptoms have been reported by people with the post-COVID-19 condition [4]. Although the exact mechanism is not entirely clear, proposed contributors include dysregulated immunity, prolonged inflammation, and direct cellular injury [4,5,6,7]. The etiology of laryngeal manifestations likely involves ongoing inflammation beyond the acute phase. SARS-CoV-2 enters respiratory epithelial cells via ACE-2, expressed in the upper airway. Evidence for viral persistence continues to accumulate, including the detection of SARS-CoV-2 proteins in plasma months after infection [8] and viral RNA reservoirs in tissue biopsies [9,10]. Higher early viral activity has been associated with an increased likelihood of developing the post-COVID condition [6,11]. Post-mortem reports have also described chronic tracheal inflammation with persistence of virus in the upper respiratory tract [12,13].

Long-term health consequences span a wide variety of symptoms, including cognitive impairment, cough, myocardial inflammation, orthostatic tachycardia syndrome, memory loss, tinnitus, and deep vein thrombosis [2,4,14]. Among reported otolaryngological manifestations, the most common are anosmia, tinnitus, sensorineural hearing loss, and dizziness [15,16].

Dysphonia has emerged as a prevalent and long-lasting symptom [17,18,19]. From 244 screened patients, 14 who had dysphonia as their only symptom were selected.

Accurate diagnosis and management of voice disorders depend on precise visualization of the larynx and objective assessment of phonation. To our knowledge, the present study represents the first report that provides detailed analyses of vocal fold parameters using high-speed videoendoscopy in post-COVID patients.

The aim is to objectively evaluate pre–post treatment changes in vocal fold oscillatory characteristics using high-speed videoendoscopy in patients with post-COVID-19 dysphonia.

## 2. Materials and Methods

### 2.1. Study Design and Setting

This was a single-center observational pre–post study conducted at the Department of Otolaryngology, Head and Neck Oncology, Medical University of Lodz, Poland. The study protocol followed the Declaration of Helsinki and was approved by the institutional Bioethical Committee (decision no. RNN/96/20/KE from 8 April 2020; and RNN/252/23/KE approved on 14 November 2023). All participants provided written informed consent prior to inclusion.

### 2.2. Participants

We screened a consecutive ambulatory cohort of adults with a documented history of SARS-CoV-2 infection and persistent laryngological complaints. Inclusion criteria were (i) age ≥18 years; (ii) symptoms consistent with dysphonia lasting ≥12 weeks after acute infection in line with post-COVID-19 condition definitions; (iii) ability to complete patient-reported outcome questionnaires. Exclusion criteria were prior laryngeal surgery; known benign or malignant laryngeal lesions; neurological voice disorders; active upper-airway infection; and professional voice overuse (to rule out previous work-related injuries).

**Figure 1:**
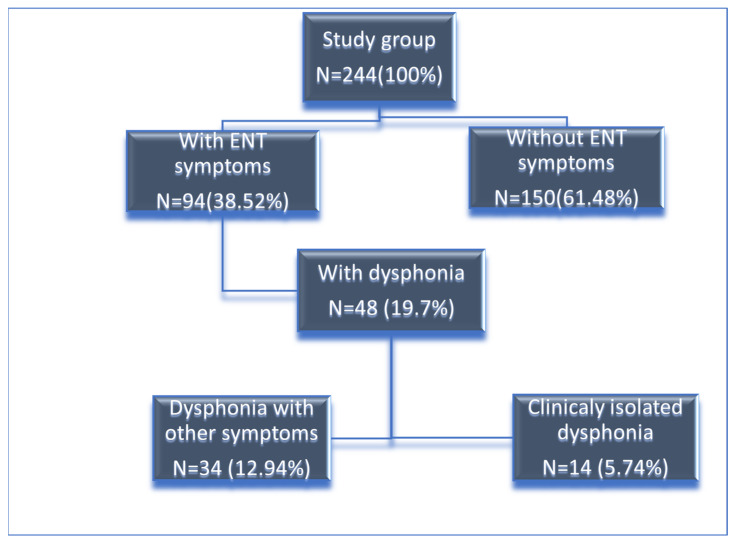
Diagram of the sample selection.

Of 244 screened patients, 14 presented isolated dysphonia and underwent standardized baseline and 6–8-week assessments (pre–post design). A CONSORT-like flow diagram of screening, exclusions, and follow-up is provided in Figure 1.

### 2.3. Patient-Reported Outcomes (PROs)

Participants completed the questionnaires Voice Handicap Index (VHI) and Voice-Related Quality of Life (V-RQOL) at baseline and at 6–8-week follow-up. Total scores and subscale scores were recorded as per instrument manuals. When one or more items were missing on a scale, the instrument’s prorating rules for missing data were applied; otherwise, the scale score was considered missing.

### 2.4. Laryngological Examination and Imaging

Videolaryngostroboscopy (VLS) and high-speed videoendoscopy (HSV) were performed by experienced laryngologists. Endoscopic examinations were carried out using a rigid 70° endoscope with white-light illumination; VLS used a stroboscopic light source synchronized to the fundamental frequency during sustained (a) at comfortable pitch and loudness. HSV recordings were acquired during the same sustained (a) with careful endoscope positioning and minimal supraglottic compression. Prior to each session, white balance and exposure were standardized.

#### 2.4.1. High-Speed Videoendoscopy (HSV) Acquisition

HSV was recorded at 3200 frames per second with resolution and exposure optimized to avoid motion blur. Each trial lasted ≥1 s of steady phonation. When multiple trials were available, the highest-quality segment free of motion with stable fundamental frequency was selected for analysis by a priori criteria. To enhance reproducibility, histogram equalization and contrast normalization were applied when required by image quality.

#### 2.4.2. Rater Blinding and Reliability

Two trained raters, blinded to timepoint (baseline vs. follow-up) and clinical data, performed quality checks and region-of-interest selection. Inter-rater reliability was quantified using a two-way random effects intraclass correlation coefficient (ICC, absolute agreement) for continuous HSV indices on a random 20% subset. Discrepancies were resolved by consensus.

#### 2.4.3. Post-Processing and Kymographic Analysis

Recordings were processed offline using dedicated software to generate kymograms, glottal width waveforms (GWW), and phonovibrograms. Objective HSV-derived indices included period- and amplitude-perturbation measures (e.g., Jitt, Jita, APF, Shimmer, ARAP, APQ3, APQ5) determined in long-term variability (LTV) analysis [20], and open quotient (OQ), amplitude measures, left–right amplitude asymmetry, phase asymmetry, and inter-fold phase difference quantified in short-term variability (STV) analysis [21]. All definitions followed previously established conventions used in our center, and explanations of individual parameters are included in Supplementary Table S1 for STV parameters and Supplementary Table S2 for LTV parameters. Additionally, their description and application can be found in the publications mentioned.

### 2.5. Treatment Protocol

Methylprednisolone short-term systemic corticosteroids, inhaled corticosteroids, hyaluronic-acid inhalations, and structured voice therapy delivered by a speech-language pathologist were administered by clinical judgment.

### 2.6. Outcomes

The primary outcome was the pre–post change in a priori selected HSV parameters sensitive to glottal insufficiency (e.g., OQ or left-right phase asymmetry). Secondary outcomes included changes in other HSV indices and changes in PROs (VHI, V-RQOL).

### 2.7. Statistical Analysis

Analyses were performed in STATISTICA.PL, version 13.3 (Statsoft, Cracov) and Microsoft Excel, version 2508 (Microsoft Corporation, Redmond, WA, USA). Normality was assessed with the Shapiro–Wilk test. Depending on distribution, pre–post comparisons used paired *t*-tests or Wilcoxon signed-rank tests. For multiple HSV indices, the false discovery rate was controlled (Benjamini–Hochberg). We report effect sizes (Cohen’s d for parametric tests; rank-biserial r for non-parametric) with 95% confidence intervals. Significance was set at α = 0.05 (two-tailed). Minimal clinically important differences (MCID), where available for PROs, were considered in interpretation. Missing data were not imputed; analyses used available cases.

## 3. Results

### 3.1. Patient Overview

Between March 2021 and October 2024, we screened 244 post-COVID outpatients. Of these, 150 were excluded due to non-laryngeal primary complaints, 8 due to structural laryngeal lesions, and 7 for incomplete records. The process of selecting the study sample is shown in Figure 1. A total of 14 patients presented isolated dysphonia and completed both baseline and 6–8-week assessments (pre–post set). The 14 patients had a median age of 51 years (IQR 43.75–70), 8 women; 6 men, 5 current/former smokers; median time from acute SARS-CoV-2 infection to baseline 15 weeks. Since we investigated the differences between pre- and post- parameters for each patient separately, we did not divide the group by gender.

### 3.2. Subjective Voice Assessment

All patients reported moderate-to-severe voice handicap at baseline: no pre-treatment VHI total score was <30, and the highest score was 88. No participant had a post-treatment VHI total >60. Paired *t*-tests showed significant improvements in the VHI total and in each VHI domain (functional, physical, emotional; *p* < 0.05). With the biggest improvement seen in functional subscale.

**Figure 2:**
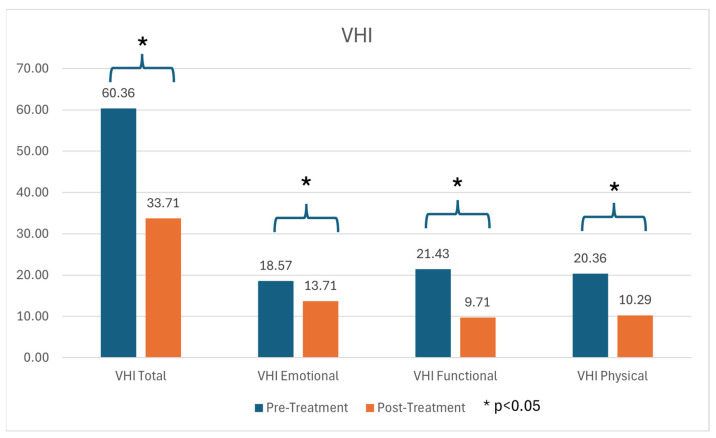
Pre- and post-treatment Voice Handicap Index (VHI) total and domain scores. Statistics: paired *t*-test; display as mean ± SD; *p* < 0.05.

The mean VHI total decreased from 60.36 (IQR 53.25–60.01) pre-treatment to 33.71.

(IQR 30.5–38) post-treatment (see Figure 2).

The mean V-RQOL increased from 55 pre-treatments to 80 post-treatments, indicating a clinically meaningful improvement in voice-related quality of life. Patients reported improved ability to speak clearly and reduced social withdrawal. Overall, self-assessment measures demonstrated significant within-patient and group-level improvements in voice function and quality of life after treatment.

### 3.3. Objective Voice Assessment

We assessed vocal fold oscillation parameters evaluating values pre- and post-treatment. A total of 23 parameters were measured—11 reflecting short-term variability (STV) and 12 representing long-term variability (LTV).

Significant differences between pre- and post-treatment values were found for 4 out of 11 STV parameters: AmpAvg, AmpAvg_2/3, AmplAsymAvg, AbsPhaseDiffAvg. Both parameters indicating amplitude showed a significant increase in post-treatment, while the two parameters describing asymmetry and phase difference demonstrated a significant decrease following treatment. Results of statistical analysis for STV parameters are shown in Table 1.

Significant differences were noted in 7 out of 12 LTV parameters: Jitt, Jita, APF, Shimmer, ARAP, APQ3, and APQ5. Values of all the parameters decreased after treatment. The pre-treatment value of average fundamental frequency F0 was incorrectly increased regardless of gender, due to hyperfunctional phonation, e.g., F0 of male Patient 1, described in detail below, was pathologically high in the initial examination. After the inflammation of the glottis was cured, the symptoms of hyperfunctional dysphonia were observed to subside in all subjects, which resulted in a decrease in the average F0 value. Results of statistical assessment for LTV parameters are shown in Table 2.

### 3.4. Illustration of Treatment Process—Representative Case

**Figure 3:**
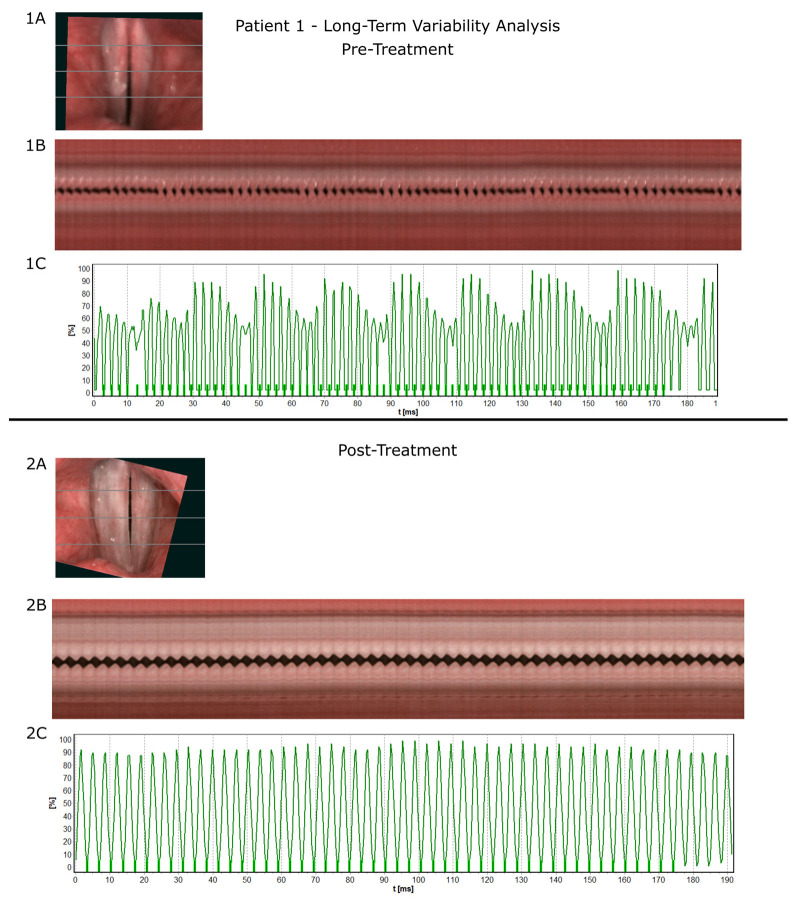
Long-term variability kymographic analysis for Patient 1, presenting results before and after treatment. Image of the glottis—(**1A**,**2A**); with gray lines marking generated kymographic cross-sections for representative thirds of glottal length. (**1B**,**2B**)—Kymograms generated for middle third part of the glottis—showing visual representation of subsequent vocal oscillations in selected cross-section. (**1C**,**2C**)—Glottal Width Waveform (GWW)—a graph visualizing changes in glottal width (*y*-axis) in function of time (*x*-axis).

We selected one of the patients from our studied group (Patient 1) as a representative case to illustrate treatment-related changes in vocal fold morphology and function. Baseline visualization showed features consistent with inflammation—erythema of the vocal folds and mucus pooling within the glottis. On LTV analysis, the mid-membranous kymogram revealed cycle-to-cycle irregularity with marked variability in both frequency and amplitude; these disturbances were corroborated by the GWW (Figure 3(1A–1C)).

After one month of treatment, there was a visible reduction in mucus accumulation and erythema. Both the kymogram and the GWW demonstrated greater cycle regularity and increased oscillation amplitude; a mild glottic insufficiency persisted (Figure 3(2A–2C)).

**Figure 4:**
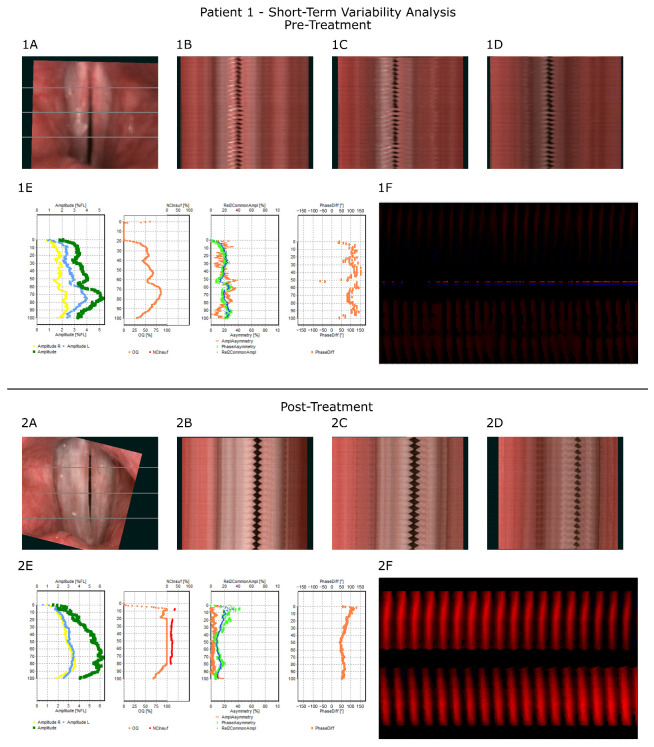
Short-term variability analysis for Patient 1, presenting results before and after treatment. A–D: the image of the glottis (**1A**,**2A**) with gray lines presenting three representative kymographic cross-sections for three functional parts of the glottis (from left to right) consecutively posterior (**1B**,**2B**), middle (**1C**,**2C**), and anterior (**1D**,**2D**) part. (**1E**,**2E**): STV analysis presented in the form of diagrams, along the glottal axis (*y*-axis on each diagram), from left to right: amplitude measures, glottal dynamic characteristics (Open Quotient), and two diagrams presenting symmetry measures (first: amplitude and phase asymmetry; second: phase difference). (**1F**,**2F**)—Phonovibrogram—a diagram presenting the movement of the vocal folds in time in relation to the center of the glottis—the center of the diagram indicates the anterior part of the glottis, upper part describes the movement of the left vocal fold while lower part describes the movement of right vocal fold—darker color indicates decreased amplitude (as described in the text).

At baseline, the plots showed increased amplitude and phase asymmetry and a larger inter-fold phase difference, together with a decrease in oscillation amplitude, mostly in the right vocal fold. The phonovibrogram exhibited overall darkening, consistent with low oscillatory amplitude (Figure 4(1A–1F)).

At post-treatment assessment, vocal fold morphology showed clear improvement, as noted above. The plots demonstrated an overall increase in oscillation amplitude and reductions in both asymmetry and phase difference. The previously noted glottic insufficiency was also reflected in the open-quotient trace (baseline), with a subsequent decrease after treatment. The phonovibrogram displayed more regular glottal cycles with higher amplitude (Figure 4(2A–2F)).

In the final step of the qualitative review, the clinician juxtaposed the pre- and post-treatment parameter sets. The tables below report the values analyzed for Patient 1.

For long-term variability (LTV) indices (Table 3), a decrease across all jitter and shimmer measures was observed, accompanied by a lower mean fundamental frequency. For short-term variability (STV) metrics (Table 4), amplitude-based measures increased, whereas the amplitude/phase asymmetry and inter-fold phase difference decreased; the open quotient also increased. These numerical findings mirror the qualitative inspection and diagram interpretation.

## 4. Discussion

In this study, we presented a detailed parametric, instrumental assessment of voices in patients with post-COVID-19 dysphonia. Our findings have implications for the clinical evaluation and monitoring of post-COVID dysphonia, particularly for documenting treatment response with objective measures.

In our outpatient cohort, 19.7% of patients experienced dysphonia, and 5.74% presented isolated dysphonia following mild-to-moderate COVID-19. These results are consistent with recent reports on the prevalence of dysphonia in post-COVID cohorts [22,23]. Beyond dysphonia, several studies have highlighted ENT manifestations in relation to the COVID-19 pandemic, including a reduced incidence of otitis media with effusion, likely linked to mask use and social restrictions [24]. In studies using instrumental methods, authors similarly described asymmetric mucosal waves, irregular periodicity, reduced amplitude, and incomplete glottic closure, which mirrors our observations [25].

Importantly, the dysphonia observed in our participants appeared primarily organic, consistent with severe corditis verified on objective examination. Other authors have identified cases of muscle tension dysphonia potentially linked to emotional distress or altered breathing patterns after the initial infection. For example, Cantarella et al. followed non-hospitalized patients by daily phone calls and reported a high rate and persistence of self-evaluated dysphonia [17,26].

Accurate diagnosis and longitudinal management of voice disorders require precise laryngeal visualization and objective phonatory assessment. The two following complementary techniques were used: videolaryngostroboscopy (VLS) and high-speed videoendoscopy (HSV). VLS is widely available and creates a slow-motion illusion by synchronizing a strobe light with the patient’s fundamental frequency, but it samples distinct phonatory cycles and depends on stable, sustained phonation, which may be difficult in severe dysphonia [27,28,29]. HSV addresses these limitations by providing true cycle-to-cycle imaging at high frame rates, enabling visualization of aperiodic events and fine-grained kymographic quantification [20,27,30]. Recent comparative data confirm that HSV yields a higher rate of recordings suitable for objective kinematic analysis than VLS in diverse voice-disorder populations [27]. Although historically used mainly in functional disorders, HSV has gained importance for organic glottic lesions as well [21,31]. Accordingly, HSV may be prioritized when subtle or aperiodic oscillations are suspected [28,32].

In our HSV recordings of patients with post-COVID dysphonia, we observed features consistent with corditis, glottic insufficiency, and reduced pliability of the vocal folds. These qualitative findings were corroborated by quantitative perturbation and periodicity indices. After approximately one month of combined pharmacological therapy and voice rehabilitation, we documented increased oscillation amplitude and reduced asymmetry/phase difference, indicating partial restoration of vibratory function. Early, targeted intervention may help mitigate chronicity, restore communication abilities, and reduce social withdrawal.

Our results also align with the known behavior of HSV-derived parameters [33,34]. Long-term variability measures (e.g., jitter, shimmer) reflect cycle stability in frequency and amplitude and are sensitive to inflammatory edema [35]. In our cohort, Jitt, Jita, APF, Shimmer, ARAP, APQ3, and APQ5 were elevated at baseline and decreased after treatment, suggesting reduced tissue inflammation and improved muscle tone, with more physiological oscillations. Recent case–control work corroborates that cycle-to-cycle perturbation measures (e.g., jitter, shimmer) differentiate post-COVID voices from controls, aligning with our LTV findings [36].

Short-term variability metrics capture behavior over a few cycles—amplitude, open quotient, asymmetry (amplitude/phase), and inter-fold phase difference. During acute inflammation, we observed reduced amplitude, particularly at the mid-membranous segment (the usual site of maximal motion), with increased asymmetry and phase difference [37,38,39]. Following treatment, amplitude increased, and asymmetry/phase difference decreased, consistent with resolution of edema and improved vocal fold biomechanics.

Importantly, improvements on instrumental evaluation were paralleled by patient-reported outcomes (VHI and V-RQOL), indicating meaningful gains in voice-related quality of life during the observation period. Pre-treatment scores reflected substantial handicap, with post-treatment improvements supporting the clinical relevance of the objective changes.

Current reviews underscore the role of structured speech–language therapy for post-COVID dysphonia, with emerging data supporting tele-voice delivery models in selected patients [40,41].

Methodological advances suggest HSV-based stiffness mapping may further refine objective characterization of glottal biomechanics in future studies [42].

Limitations include the small sample size, single-center design, absence of a control group, and potential selection bias inherent to a tertiary-care outpatient cohort. The pre–post design also leaves room for regression to the mean and spontaneous improvement. Finally, multiple comparisons across numerous parameters may inflate type-I error; future studies should consider adjustment for multiplicity and report effect sizes with confidence intervals alongside *p*-values. Despite these limitations, our data support the feasibility and clinical utility of HSV-based assessment in post-COVID dysphonia and motivate controlled, larger-scale investigations.

## 5. Conclusions

Dysphonia is a prevalent and long-lasting symptom of the post-COVID-19 condition. Patients require urgent and thorough ENT examination, treatment, rehabilitation, and close monitoring. The HSV technique provides greater precision for reliable laryngeal evaluation and assessment of phonatory function than laryngeal videostroboscopy. Although post-COVID dysphonia can be challenging, our findings suggest that add-on multimodal short-term treatment may enable full recovery; however, larger controlled studies are needed.

## Figures and Tables

**Table 1:** Results of statistical analysis for short-term variability parameters. Boldened font indicates parameters with significant differences (*p* < 0.05). *—parameters with non-normal distribution, analyzed with the use of Wilcoxon test.

	Pre-Treatment			Post-Treatment			
Parameter	Mean	Median	SD	Mean	Median	SD	*p* value
**AmpAvg [%FL]**	6.721	5.450	4.243	9.279	7.300	5.861	**0.00850**
**AmpAvg_2/3 [%FL]**	7.257	6.400	4.789	10.200	7.950	5.920	**0.00996**
Non-opening [%FL]	2.743	0.000	5.414	1.600	0.600	3.461	0.40081 *
RGGA [%]	11.943	2.400	21.186	8.814	1.550	14.300	0.22707
OQAvg [%]	63.700	61.150	14.674	64.893	57.950	19.361	0.66035 *
OQAvg_2/3 [%]	61.807	58.200	18.688	69.550	64.850	21.301	0.06115
**AmplAsymAvg [%]**	15.521	11.650	9.545	14.443	12.900	7.883	**0.02176**
AmplAsymAvg_2/3 [%]	15.457	14.150	10.444	11.279	10.300	6.395	0.75315 *
PhaseAsymAvg [%]	16.529	12.850	12.344	16.814	18.200	10.799	0.45919
PhaseAsymAvg_2/3 [%]	16.950	10.900	15.461	18.293	16.050	14.058	0.35913
**AbsPhaseDiffAvg [°]**	63.979	62.200	25.212	48.993	52.100	26.724	**0.01142**

**Table 2:** Results of statistical analysis for long-term variability parameters. Boldened font indicates parameters with significant differences (*p* < 0.05). *—parameters with non-normal distribution, analyzed with the use of Wilcoxon test.

	Pre-Treatment			Post-Treatment			
	Mean	Median	SD	Mean	Median	SD	*p* value
F0Avg [Hz]	258.857	215.050	135.641	233.936	212.500	119.514	0.11585 *
PPF [%]	7.456	3.615	9.525	2.585	1.025	4.112	0.09609 *
**Jitt [%]**	7.651	3.715	10.256	2.469	1.020	3.817	0.03924 *
**Jita [ms]**	0.309	0.135	0.364	0.136	0.045	0.248	0.02771 *
**APF [%]**	14.336	6.415	14.146	3.946	2.360	5.959	0.01075 *
**Shimmer [%]**	12.669	6.425	11.243	3.930	2.345	5.971	0.00604 *
PRAP [%]	4.192	2.135	5.412	1.461	0.710	2.182	0.05462 *
**ARAP [%]**	7.015	3.425	6.556	2.287	1.255	3.404	0.01310 *
PPQ3 [%]	4.069	2.075	5.021	1.506	0.725	2.283	0.05462 *
PPQ5 [%]	5.186	2.380	6.701	1.767	0.820	2.667	0.07474 *
**APQ3 [%]**	7.786	3.425	7.900	2.321	1.265	3.472	0.01206 *
**APQ5 [%]**	8.844	4.110	8.657	2.420	1.360	3.559	0.00713 *

**Table 3:** Values of long-term variability parameters for Patient 1 before and after treatment.

Patient 1	Long-Term Variability Parameters	
	Pre-Treatment	Post-Treatment
F0Avg [Hz]	427.8	286.8
PPF [%]	7.56	0.27
Jitt [%]	7.37	0.27
Jita [ms]	0.17	0.01
APF [%]	26.93	1.51
Shimmer [%]	19.29	1.51
PRAP [%]	3.73	0.17
ARAP [%]	8.07	0.9
PPQ3 [%]	3.79	0.17
PPQ5 [%]	5.46	0.16
APQ3 [%]	10.41	0.9
APQ5 [%]	13.87	0.86

**Table 4:** Values of short-term variability parameters for Patient 1 before and after treatment.

Patient 1	Short-Term Variability Parameters	
	Pre-Treatment	Post-Treatment
AmpAvg [%FL]	3.8	4.9
AmpAvg_2/3 [%FL]	3.5	5.5
Non-opening [%FL]	17.1	1.7
RGGA [%]	0	18.1
OQAvg [%]	49	90.9
OQAvg_2/3 [%]	58.6	100
AmplAsymAvg [%]	19.2	4.3
AmplAsymAvg_2/3 [%]	18.5	4.4
PhaseAsymAvg [%]	18.8	14.7
PhaseAsymAvg_2/3 [%]	18.3	10.2
AbsPhaseDiffAvg [°]	107	67.5

## Data Availability

All the most relevant data generated or analyzed during this study are included in this published article. Remaining datasets used and/or analyzed during the current study are available from the corresponding author upon reasonable request.

## Supplementary Materials

The following supporting information can be downloaded at: https://www.mdpi.com/article/10.3390/jcm14196861/s1, Table S1: Precise description of individual Short Term Variability parameters.; Table S2: Precise description of individual Long Term Variability parameters.
