# Supplementary material for: Objective and Subjective Voice Outcomes in Post-COVID-19 Dysphonia: A High-Speed Videoendoscopy Pre–Post Study
Source: J Clin Med. 2025 Sep 28;14(19):6861. doi: 10.3390/jcm14196861 (PMC12525375; doi:10.3390/jcm14196861)
Supplement: Supplementary file 1 [file jcm-14-06861-s001.zip › jcm-3879191-supplementary.pdf]

Abbreviation	Full name of the parameter	Description
**Amplitude Measures**		
AmpAvg (%FL)	Average glottal gap amplitude	Indicates the average resultant amplitude of vocal fold movement for the glottal gap, it's middle third part and involved and healthy vocal fold respectively.
AmpAvg_2/3 (%FL)	Average amplitude of the middle third part of the glottis	
**Open Quotient measures**		
OQAvg (%); OQAvg 2/3	Average Open Quotient for glottal gap and middle third of the glottis	Indicates the ratio of glottal opening phase to whole length of vocal cycle – average value for the whole glottal gap and middle third part respectively. Values 0-100%. 0 meaning no opening, 100% meaning no closing phase.
RGGA	Relative Glottal Gap Area	The ratio of minimal to maximal area of the glottis during the cycle
Non-opening (%FL)	Non-opening part of vocal folds	Indicates part of the glottis without opening (points along glottal axis with OQ <1%)
**Symmetry measures**		
AmplAsymAvg (%); AmplAsymAvg_2/3 (%)	Average amplitude asymmetry for whole glottal gap and its middle third part respectively	This coefficient compares individual amplitudes of both vocal folds movement. 0% means that the movements are perfect reflections of each other – full symmetry; 100% means that there is no resultant vocal fold movement – they are moving in the same direction
PhaseAsymAvg (%); PhaseAsymAvg_2/3 (%)	Average Phase Asymmetry for whole glottal gap and its middle third part respectively	This coefficient compares the sum of individual amplitudes of vocal fold motion to the amplitude of their resultant movement. 0% means that the resultant movement is a perfect sum of both folds movement; 100% means that there is no resultant movement.
AbsPhaseDiffAvg (°)	Average absolute phase difference	Absolute mean value of phase difference for whole vocal folds.

Supplementary Table 1: Precise description of individual Short Term Variability parameters.

Abbreviation	Full name of the parameter	Description
F0Avg (Hz)	Average fundamental frequency	Average frequency of vocal oscillations in the sample
**Period Perturbation Measures** Parameters based on Glottal Width waveform measuring perturbation in the period length between different vocal oscillation cycles.		
Jita (ms)	Mean Jitter value given in miliseconds	Jitter represents the relative difference in length between adjacent base periods, averaged over the length of the entire sample.
Jitt (%)	Mean Jitter value given in %	
PPF (%)	Period Perturbation Factor	Coefficient representing period disturbances
PRAP (%)	Period Relative Average Perturbation	Parameter representing mean period disturbances
PPQ3 (%)	Period Perturbation Quotient with averaging area of 3 vocal cycles	Parameters representing a quotient of period disturbances with specific averaging areas over the length of the entire sample.
PPQ5 (%)	Period Perturbation Quotient with averaging area of 5 vocal cycles	
**Amplitude Perturbation Measures** Parameters based on Glottal Width waveform measuring perturbation in the amplitude between different vocal oscillation cycles.		
Shimmer (%)	Mean Shimmer value given in %	Shimmer represents the relative difference in amplitude between adjacent base periods, averaged over the length of the entire sample.
APF (%)	Amplitude Perturbation Factor	Coefficient representing amplitude disturbances
ARAP (%)	Amplitude Relative Average Perturbation	Parameter representing mean amplitude disturbances
APQ3 (%)	Amplitude Perturbation Quotient with an averaging area of 3 vocal cycles	Parameters representing a quotient of amplitude disturbances with specific averaging areas over the length of the entire sample.
APQ5 (%)	Amplitude Perturbation Quotient with an averaging area of 5 vocal cycles	

Supplementary Table 2: Precise description of individual Long Term Variability parameters.
